# Deep Eutectic Solvent-Assisted Ultrasonic Extraction of Anthocyanins from Blueberry Pomace: Optimization, Mechanistic Insights and In Vitro Antioxidant Activity

**DOI:** 10.3390/molecules31132356

**Published:** 2026-07-03

**Authors:** Lina Chen, Yue Mi, Xing Yang, Yunmei Ma, Chunting Zhu, Jing Xu, Dongfang Shi

**Affiliations:** 1 College of Chemistry, Changchun Normal University, Changchun 130032, China; chenlina4321@163.com; 2 Institute of Innovation Science and Technology, Changchun Normal University, Changchun 130032, China; 18097730680@163.com (Y.M.); yangyx0816@163.com (X.Y.); yunmei0205@163.com (Y.M.); zct15948840694@163.com (C.Z.); xujingl@yeah.net (J.X.); 3 College of Life Science, Changchun Normal University, Changchun 130032, China

**Keywords:** deep eutectic solvents, anthocyanin extraction, response surface methodology, antioxidant activity, COSMO-RS

## Abstract

This study explores an innovative approach based on deep eutectic solvent (DES)-synergistic ultrasonic-assisted extraction (UAE) of anthocyanins from blueberry pomace (BP). Choline chloride–lactic acid (ChCl-LA, 1:2) was identified as the most efficient DES and provided the highest anthocyanin recovery. The optimal conditions for ChCl-LA-synergistic UAE (ChCl-LA-UAE) were obtained as follows: ultrasonic power of 270 W, extraction time of 30 min, liquid-to-solid ratio of 35:1, and water content in ChCl-LA of 40%, achieving a total anthocyanin content (TAC) of 3.5168 mg/g, with R^2^ = 0.9860. This value was significantly higher than those obtained with 70% ethanol (3.1962 mg/g) and pure water (1.9137 mg/g). SEM images revealed that ChCl-LA-UAE disrupted the surface structure of the samples, thereby promoting anthocyanin release. COSMO-RS simulations confirmed that ChCl-LA significantly enhanced the interaction between the solvent and the representative anthocyanin (cyanidin-3-O-glucoside, C3G), resulting in higher extraction efficiency. In vitro antioxidant assays further demonstrated that the synergistic system exhibited stronger antioxidant activity. Overall, ultrasonic-synergistic DES extraction could be an eco-friendly method for recovering high-value compounds from blueberry and its byproducts.

## 1. Introduction

Blueberry (*Vaccinium* spp.), native to North America, is widely cultivated in cool, sunny climates. Recognized by the Food and Agriculture Organization (FAO) as one of the “top five healthy berries in the world” [1], it has gained global popularity due to its high economic value and medicinal potential [2,3]. Blueberry pomace (BP), the main by-product generated after further processing such as juicing, winemaking, or jam production, typically consists of peels, residual pulp, seeds, and partially disrupted cell tissues [4,5]. With the rapid development of the blueberry processing industry, the substantial generation of pomace has drawn widespread attention to its resource utilization [6,7].

Anthocyanins are water-soluble natural pigments widely distributed in plants, with particularly high contents in dark-colored fruits and vegetables such as blueberries, mulberries, and black wolfberries [8]. Due to the presence of unpaired electrons in their molecular structure, anthocyanins can donate electrons and hydrogen to free radicals, thereby effectively terminating reactive oxygen species (ROS) in the human body [9]. Under certain experimental conditions, the antioxidant capacity of anthocyanins has been reported to reach levels up to 50 times that of vitamin E (VE) and 200 times that of vitamin C (VC) [10], making them one of the natural plant components with the strongest antioxidant effects discovered so far. Studies have shown that anthocyanins also exhibit multiple physiological activities, including anti-cancer, anti-inflammatory [11], anti-aging, anti-obesity, brain function improvement, vision protection, and cardiovascular protection [12,13].

As the most important class of compounds in BP, anthocyanins endow the pomace with a deep purple to blue–black color and serve as a crucial source of its functional value [14]. The anthocyanins in BP mainly include cyanidin-3-O-glucoside, delphinidin, cyanidin, petunidin, peonidin, and malvidin, most of which exist in the form of glycosides [15]. During pomace processing, part of these anthocyanins remains in the peel and cell wall structures; especially after juicing, a large amount of anthocyanins still remain in the solid phase of the pomace, making it a high-quality raw material for anthocyanin extraction [16]. In addition to their high content, the anthocyanins in BP often form complexes with macromolecules such as pectin, cellulose, and proteins, which affects their extraction efficiency and bioavailability [17,18]. Therefore, investigating the extraction process of anthocyanins from BP is of great significance for the development and utilization of BP.

Traditional extraction methods for anthocyanins from BP mainly adopt acidified systems of organic solvents, which not only pose potential hazards to operators and the environment but also increase the costs associated with solvent recovery and residual control [19]. Furthermore, conventional extraction methods are unable to effectively break the binding interactions between anthocyanins and macromolecules such as pectin and cellulose, resulting in a low dissolution rate of anthocyanins [20,21]. In addition, anthocyanins are highly sensitive to heat, light, and oxygen during traditional extraction processes; prolonged heating under reflux tends to accelerate their degradation and affect the biological activity of the extracted products [22].

In recent years, deep eutectic solvents (DESs), as a new type of green solvent, have shown significant advantages in the extraction of anthocyanins from pomace [23,24]. DESs are usually formed by hydrogen bond acceptors (e.g., choline chloride) and hydrogen bond donors (e.g., urea, organic acids, carbohydrates) through hydrogen-bonding interactions, and they possess characteristics such as biodegradability, low volatility, non-toxicity or low toxicity, and simple preparation [25]. More importantly, the hydrogen bond network in DESs can effectively compete with and disrupt the hydrogen bond interactions between anthocyanins and polysaccharides in the cell wall of pomace, thereby significantly improving the extraction efficiency of anthocyanins [26]. Meanwhile, DES systems can enhance the stability of anthocyanins during the extraction process, slow down their degradation rate, and help obtain extracts with higher activity [27,28].

Ultrasound-assisted extraction (UAE) technology generates a “cavitation effect” in liquid media through ultrasound, which instantly produces extremely high local pressure and temperature, thereby exerting strong mechanical shear force on plant cell walls and causing their rupture [29]. This physical fragmentation significantly promotes the penetration of solvents into the matrix and the diffusion of target compounds into the solvents, thereby greatly improving extraction efficiency, shortening extraction time, and reducing solvent dosage. Bamba et al. [11] specifically optimized the process of UAE for extracting polyphenols from BP, confirming its significant advantages over traditional methods. Hu et al. [30] further confirmed the synergistic effect of ultrasound in extracting anthocyanins from BP. At present, DESs and UAE have been applied to the extraction of plant anthocyanins. However, research on BP, a material with a complex matrix, remains limited. Previous studies on DES-based extraction of anthocyanins have largely focused on optimizing extraction conditions, while molecular-level investigations of solvent–solute interactions remain relatively limited. In particular, the combination of COSMO-RS calculations with experimental DES screening for BP anthocyanins has rarely been reported.

In this work, an efficient DESs-UAE integrated method was explored for the extraction of anthocyanins from BP, and the extraction conditions were optimized by both single factor experiments and response surface methodology (RSM). The interaction of the representative anthocyanin (C3G) with different solvent systems was investigated via COSMO-RS analysis. In addition, the microscopic mechanism of the optimal DES was also elucidated using COSMO-RS. Finally, a comparative study of the extraction effects and in vitro antioxidant activities between BP extracts obtained using DES-UAE synergistic extraction and conventional methods was performed.

## 2. Results and Discussion

### 2.1. Thermodynamic Screening Based on lnγ

DESs are composed of hydrogen bond acceptors (HBAs) and hydrogen bond donors (HBDs). The specific types and molar ratios of these components directly dictate the physicochemical properties of the system, such as polarity, viscosity, and solubility. To systematically compare the structural differences between quaternary ammonium and zwitterionic compounds, choline chloride (ChCl) and betaine (Bet) were selected as HBAs. For HBDs, five compounds with diverse functional groups were chosen, including lactic acid (monocarboxylic with hydroxyl), DL-malic acid (dicarboxylic with hydroxyl), citric acid (tricarboxylic with hydroxyl), oxalic acid (dicarboxylic without hydroxyl), and 1,4-butanediol (diol without carboxyl). This selection was driven by several key considerations: the green, safe, and biocompatible nature of ChCl and Bet for food-related applications; the strong hydrogen-bonding capacity and tunable acidity of organic-acid HBDs, which benefit anthocyanin stability and extraction efficiency; the use of 1,4-butanediol to assess the role of hydroxyl groups in the absence of carboxyl groups; and the proven feasibility of similar DES systems from previous studies, further supported by our preliminary experiments. Accordingly, seven DES systems (Table 1) were prepared.

**Figure 1:**
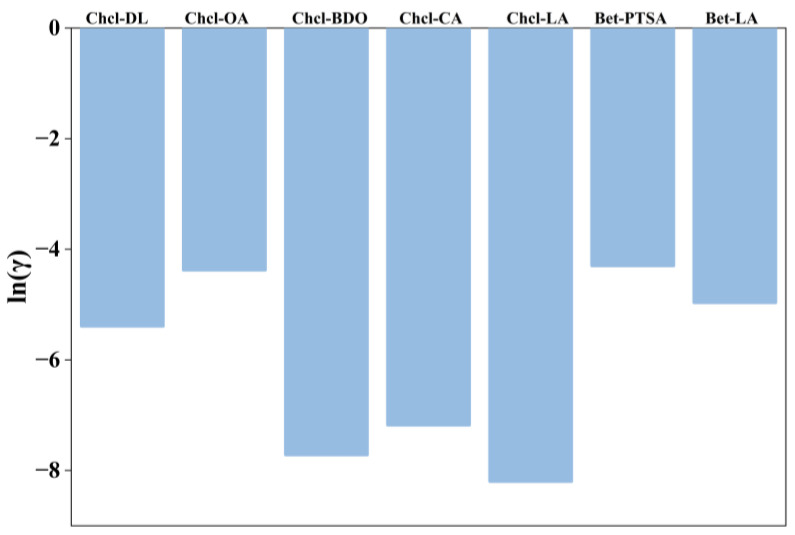
Activity-based screening of different solvent types.

To evaluate the dissolution capacity of these DESs for cyanidin-3-O-glucoside (C3G), the infinite dilution activity coefficients ln(γ) were calculated using the COSMO-RS model (Figure 1). The ln(γ) values ranged from −8.197 to −4.298 (range of 3.899), confirming that DES composition decisively affects dissolution performance. DES-5 (ChCl/lactic acid, 1:2) showed the lowest ln(γ) value (−8.197), indicating optimal solubility for C3G, followed by DES-3 and DES-4. Conversely, DES-6 (betaine/PTSA·H_2_O, 1:1) exhibited the highest ln(γ) value (−4.298), corresponding to the poorest solubility. This ranking provides a clear thermodynamic basis for subsequent microscopic mechanism analysis.

### 2.2. Screening of Different DESs

**Figure 2:**
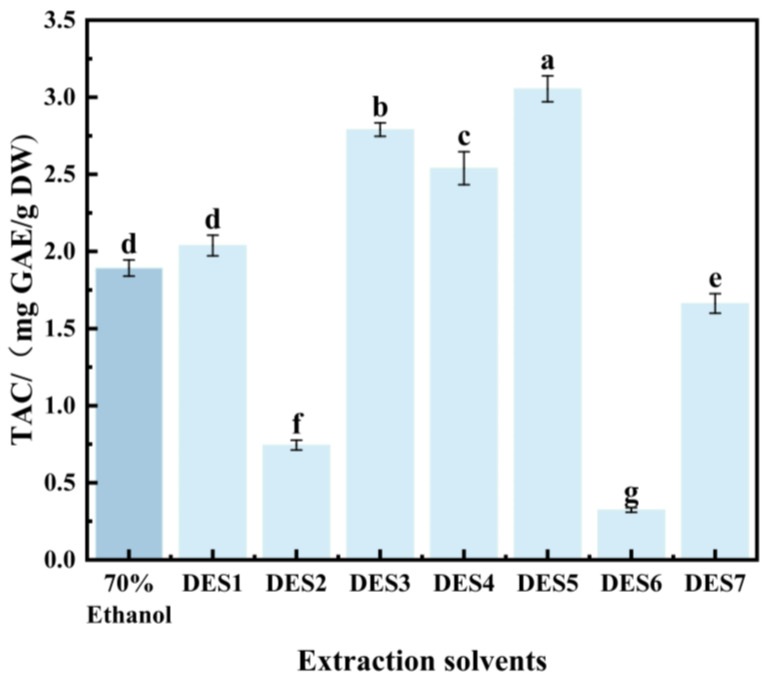
Effect of different solvent types on the TAC of BP. a–g: Significant differences among solvent types at *p* < 0.05.

To verify the reliability of the COSMO-RS-based computational screening, a series of choline chloride- and betaine- based DESs (Table 2) were prepared and used to extract anthocyanins from BP. To reduce viscosity, all DESs were adjusted to 30% (*v*/*v*) water prior to extraction. A 70% (*v*/*v*) ethanol solution was used as a reference solvent. Under identical extraction conditions (40 °C, 30 min, solid-to-liquid ratio of 1 g:25 mL, and 30% water content), seven DESs outperformed 70% ethanol in anthocyanin extraction, as shown in Figure 2. Among them, DES-5 (choline chloride/lactic acid, 1:2) yielded the highest TAC of 3.0545 mg/g, which was highly consistent with the ln(γ) calculation results. Thus, the COSMO-RS-based screening method demonstrated good predictive ability, and DES-5 was identified as the optimal solvent for subsequent extraction.

### 2.3. Characterization of DES Systems

#### 2.3.1. FTIR Analysis of DES Systems

**Figure 3:**
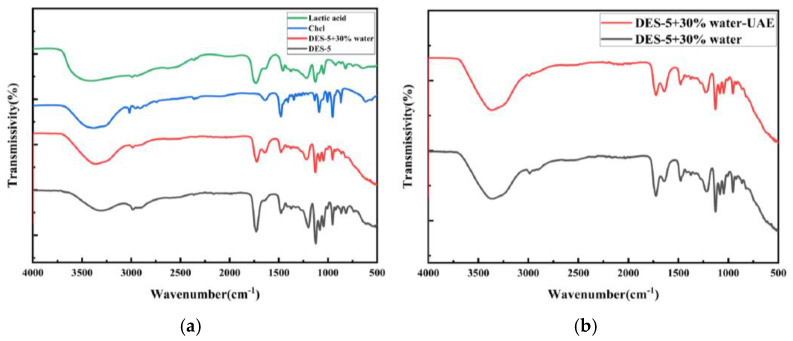
FTIR spectra of (**a**) ChCl, LA, DES-5, and DES-5 with 30% water; (**b**) DES-5 with 30% water and the DES-5 extract.

To confirm the successful formation of the seven DES systems and investigate the effect of water addition, FTIR spectra of pure DESs and DESs with 30% water were recorded. The spectra of pure ChCl, LA, DES-5 with 30% water, and the DES-5 extract are presented in Figure 3 (FTIR spectra of the other six DES systems are provided in Figure S1). As shown in Figure 3a, upon DES formation, the O–H stretching band of LA at 3400 cm^−1^ broadened and shifted to 3250 cm^−1^, while the C=O stretching band shifted from 1720 cm^−1^ to 1705 cm^−1^, indicating hydrogen bonding between ChCl and LA [31] and confirming successful DES synthesis. With 30% water addition, the O–H band further broadened and shifted to 3350 cm^−1^, and the C=O band shifted to 1715 cm^−1^, suggesting that water participated in the hydrogen-bonding network without disrupting the DES structure, consistent with the optimal water content identified in the extraction experiments. Figure 3b compares the FTIR spectra of DES-5 with 30% water and the DES-5 extract. The extract exhibited significantly lower transmittance in the O–H stretching region (3200–3600 cm^−1^) and the C=O stretching region (1600–1700 cm^−1^), indicating the presence of anthocyanins and other phenolic compounds extracted from BP. The stronger absorption bands for the extract than for the pure DES-5 solution confirmed the successful extraction of anthocyanins into the DES system. Overall, FTIR analysis confirmed the successful synthesis of DES-5, the stability of its hydrogen-bonding network upon 30% water addition, and the effective extraction of anthocyanins from BP.

#### 2.3.2. The pH of DES Systems

The pH values of DES systems are summarized in Table 2. As can be seen, no direct correlation was observed between pH and the extraction yield under the present experimental conditions. For example, DES-2 exhibited the lowest pH (0.04) but relatively low extraction efficiency, while DES-5 (pH 1.22) provided the highest TAC. This suggests that excessively strong acidity may lead to anthocyanin degradation, whereas moderate acidity favors anthocyanin stability and extraction. Among the two lactic acid-based DESs, DES-5 (ChCl/LA, 1:2, pH 1.22) outperformed DES-7 (Bet/LA, 1:1, pH 3.69), indicating that ChCl-based DESs provide stronger acidity than betaine-based systems. Overall, extraction efficiency is determined by the combined effects of pH, hydrogen-bonding capacity, and viscosity, rather than pH alone. DES-5 achieved the optimal balance among these factors, resulting in the highest extraction efficiency.

### 2.4. Optimization of DES-5

**Figure 4:**
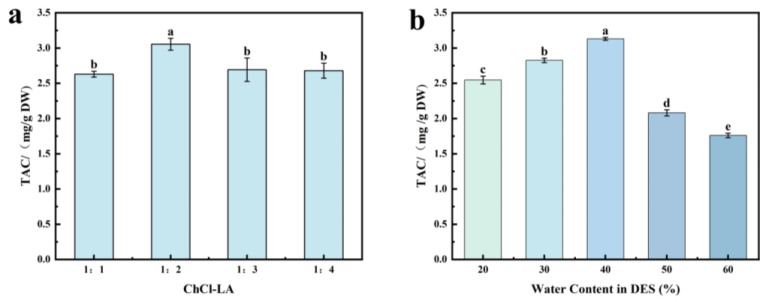
Optimization of DES: (**a**) effect of different molar ratios of DES-5 on the TAC of BP, (**b**) effect of different water contents of DES-5 on the TAC of BP. Different lowercase letters indicate significant differences among groups (*p* < 0.05, ANOVA).

Different compounds exhibit distinct polarity distributions and hydrogen bond sites. Adjusting the molar ratio of DES can fine-tune its physicochemical properties to achieve optimal compatibility with the target compound. Therefore, the molar ratio of the choline chloride–lactic acid system was further optimized. As shown in Figure 4a, the highest anthocyanin extraction efficiency was achieved when the molar ratio of HBA to HBD was 1:2. Consequently, the choline chloride–lactic acid system with a molar ratio of 1:2 was selected for subsequent experiments.

Although DESs exhibit excellent extraction performance, their high viscosity remains a major limitation for widespread application. Previous studies have shown that the addition of a small amount of water not only reduces DES viscosity but also expands the hydrogen bond network and increases solvent polarity. However, water addition must be strictly controlled, as excess water (>50% *v*/*v*) disrupts the DES hydrogen bond network, reducing its affinity for the target analyte and decreasing extraction efficiency [32]. Therefore, the optimal water content for the choline chloride–lactic acid (1:2) system was systematically investigated. As shown in Figure 4b, the extraction efficiency increased with water content, reached a maximum at 40% (*v*/*v*), and then decreased. This trend is consistent with previous reports that excess water disrupts DES hydrogen bonds, thereby reducing extraction efficiency.

### 2.5. Microscopic Mechanism Underlying the Optimal Performance of DES-5

To further explain the microscopic reasons for the superior dissolution performance of DES-5, three aspects were analyzed: σ-profile hydrogen-bonding compatibility, IGMH weak interactions, and ESP electrostatic potential.

#### 2.5.1. σ-Profile Hydrogen-Bonding Compatibility Analysis

**Figure 5:**
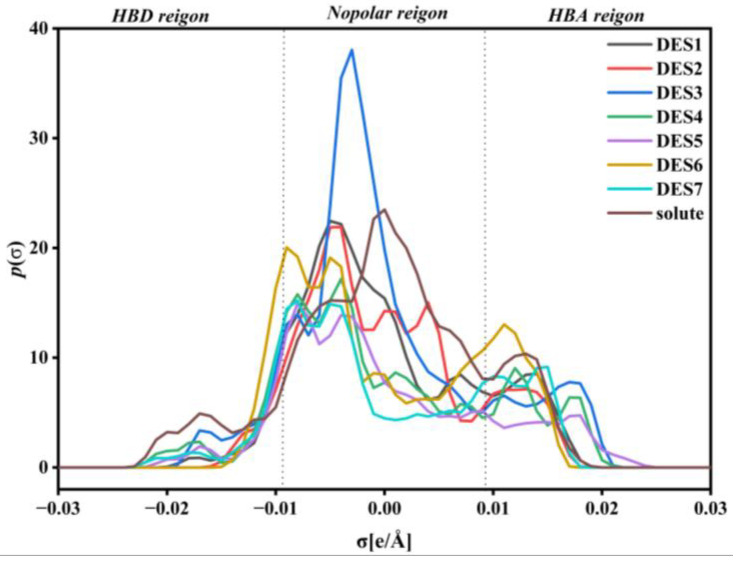
σ-profiles of the seven DESs and C3G. The dashed line indicates the baseline at σ = 0, separating the hydrogen-bond donor (HBD, negative σ) and acceptor (HBA, positive σ) regions.

A hydrogen-bonding matching score (HB_Score) was established by quantifying three dimensions: HBA strength, HBD strength, and molecular steric compatibility. Each component was scored from 0 to 10 based on the σ-profile peak intensities (for HBA and HBD strength) and molecular structural features (for steric compatibility). The three scores were summed with equal weighting and normalized to a 10-point scale. The results are shown in Table 1. The data showed a strong negative correlation between HB_Score and ln(γ) (Pearson correlation coefficient r ≈ −0.94), supporting the effectiveness of this scoring system. The σ-profiles of the seven DESs and C3G are shown in Figure 5.

#### 2.5.2. IGMH Visualisation Analysis of Weak Interactions

**Figure 6:**
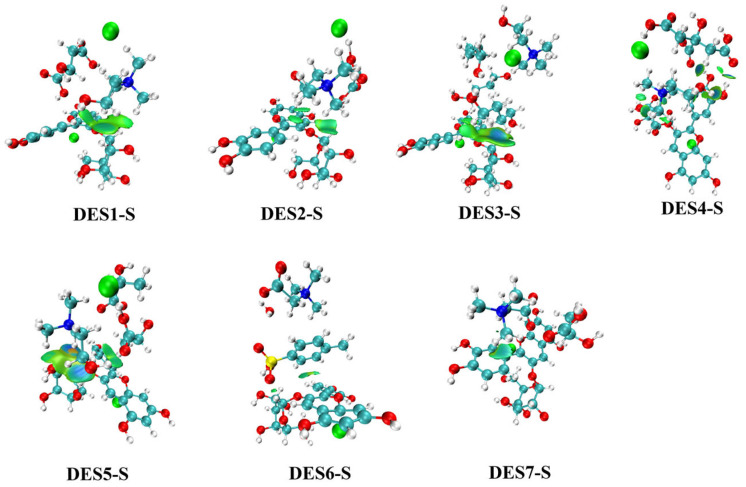
IGMH isosurface maps of C3G-DES complexes.

Cluster models of C3G with each DES were constructed and subjected to IGMH analysis (Figure 6), where green isosurfaces represent attractive interactions (mainly hydrogen bonds) and blue isosurfaces represent steric hindrance. In the DES-5 complex, dense and widely distributed green isosurfaces were observed between C3G and DES-5, covering multiple hydrogen-bonding sites and forming a bridge-like interaction network. DES-3 and DES-4 also exhibited relatively dense green isosurfaces, though to a lesser extent. In contrast, the DES-6 complex showed significantly fewer green isosurfaces with blue regions at the solute–solvent interface, possibly due to the steric hindrance of PTSA. DES-7 displayed limited green isosurfaces, likely related to the weaker HBA ability of betaine. The green isosurface densities of DES-1 and DES-2 complexes were between those of DES-4 and DES-7, consistent with their moderate solubility (lnγ of −5.388 and −4.376, respectively). This gradient change suggests a positive correlation between green isosurface density and solubility. Overall, the IGMH analysis provided molecular-level evidence that DES-5 formed a stronger hydrogen-bonding network with C3G, whereas the attractive interactions of low-solubility DESs (especially DES-6) were weak and accompanied by steric repulsion.

#### 2.5.3. ESP Electrostatic Potential and Surface Charge Quantitative Analysis

**Figure 7:**
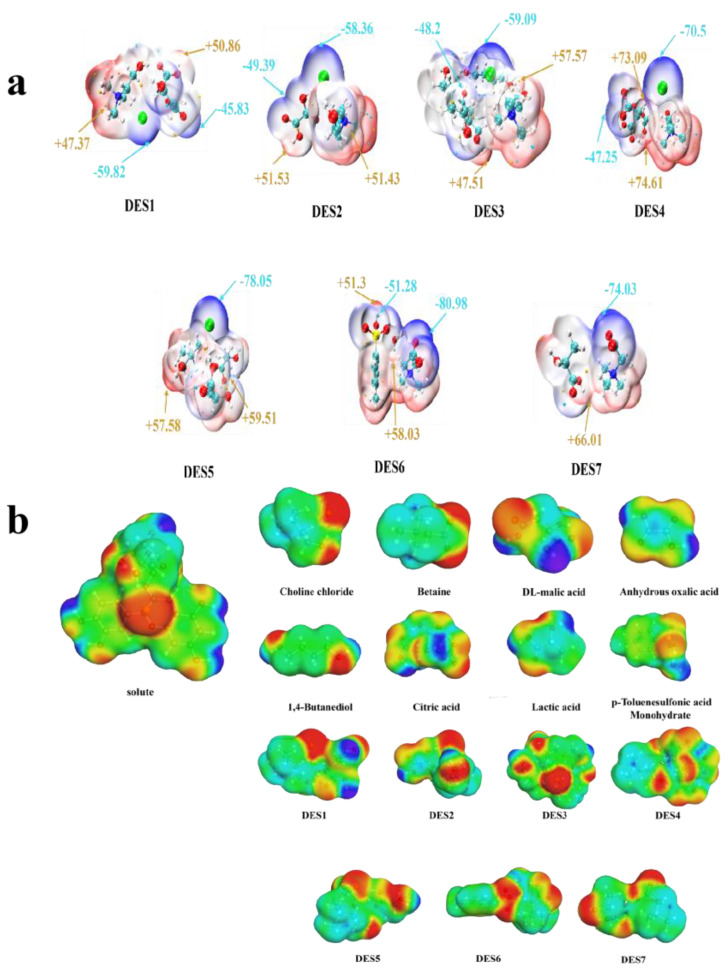
ESP and surface charge quantitative analysis of C3G and DESs. (**a**) Partial atomic charges of key interacting atoms for C3G and DES−1 to DES−7. (**b**) Molecular structures of DES components.

Molecular surface electrostatic potentials and partial atomic charges of key atoms were calculated using DFT (Figure 7). The ESP distribution of C3G showed concentrated negative regions, with the most negative oxygen atom at −64.26 (Figure 7a). For DES-5, the hydroxyl hydrogen of lactic acid exhibited a charge of +57.58, yielding an electrostatic potential difference Δ of 121.84 with C3G, suggesting strong electrostatic attraction. Notably, DES-4 and DES-7 showed higher donor charges (+57.57 and +58.03) and larger Δ values (121.83 and 122.29), yet lower solubility than DES-5. This indicates that electrostatic potential difference alone does not determine solubility. A possible explanation is that the rigid tricarboxylic structure of citric acid in DES-4 may reduce donor site accessibility, while the replacement of choline chloride with betaine in DES-7 may weaken overall HBA ability. DES-6, which exhibited the poorest solubility, had a donor charge of +51.30 (Δ = 115.56), likely due to its weak HBA ability and the steric hindrance of PTSA. In summary, the ESP and surface charge analysis suggests that DES-5 achieves a favourable balance among HBA strength, HBD accessibility, and steric compatibility, providing quantitative support for its superior hydrogen bond strength.

### 2.6. Results of the One-Factor Experiment

#### 2.6.1. Influence of the Solid-to-Liquid Ratio

**Figure 8:**
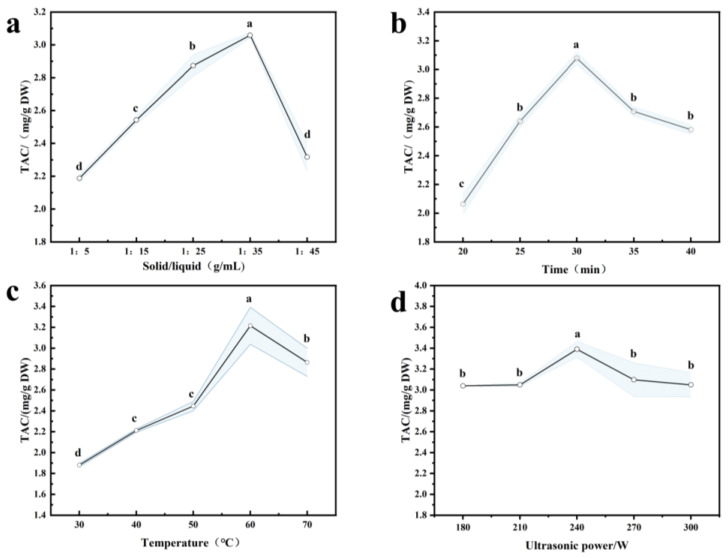
Results of single-factor experiments: (**a**) solid-to-liquid ratio, (**b**) ultrasonic time, (**c**) ultrasonic temperature, (**d**) ultrasonic power. Different letters indicate significant differences among groups at *p* < 0.05. Blue shaded areas in the figure represent the error bands.

As shown in Figure 8a, the yield of anthocyanins from BP first increased and then decreased when the solid-to-liquid ratio ranged from 1:5 to 1:45 (g/mL). The highest yield of 3.0587 mg/g was obtained at a ratio of 1:35 (g/mL). Beyond this ratio, the yield gradually decreased. This may be attributed to the increased contact area between DES and anthocyanin molecules with increasing solvent volume, which enhanced mass transfer and promoted anthocyanin dissolution. However, once the yield reached its maximum, excess DES increased mass transfer resistance, potentially damaged the anthocyanin structure, and extracted more impurities, thereby reducing ultrasonic transmission efficiency and leading to a decrease in anthocyanin yield. Therefore, 1:35 (g/mL) was finally selected as the optimal ratio, reflecting the dual effect of solvent usage on the mass transfer process.

#### 2.6.2. Influence of Ultrasonic Time

According to the results in Figure 8b, the extraction yield of anthocyanins from BP first increased and then decreased with ultrasonic treatment times ranging from 20 to 40 min. The maximum yield of 3.0782 mg/g was obtained at 30 min. After 30 min, the yield gradually decreased. This indicates that a slight increase in ultrasonic time can improve the mass transfer process, but excessive ultrasonic time causes thermal degradation. The reason may be that the amount of anthocyanins extracted gradually increased until equilibrium with DES was reached; however, beyond that point, the yield decreased due to the thermal instability of anthocyanins. Consequently, 30 min was determined as the optimal ultrasonic treatment time.

#### 2.6.3. Influence of Ultrasonic Temperature

According to the data in Figure 8c, the extraction yield of anthocyanins from BP first increased and then decreased with ultrasonic temperatures ranging from 30 to 70 °C. The highest anthocyanin content of 3.2145 mg/g was observed at 60 °C. Beyond this temperature, the yield gradually decreased. This may be explained by the increased solubility and diffusion of anthocyanins in DES at higher temperatures, which promoted their release. However, when release approached saturation, further temperature increase caused thermal degradation of anthocyanins, reducing their content. Therefore, 60 °C was determined as the optimal ultrasonic temperature.

#### 2.6.4. Influence of Ultrasonic Power

As shown in Figure 8d, the yield of anthocyanins from BP first increased and then decreased with increasing power within the range of 180–300 W. The maximum yield of 3.3899 mg/g was achieved at 240 W. Beyond this power, the yield decreased as power increased. This may be because a moderate increase in power enhanced cavitation and mechanical vibration effects, breaking the cell structure and promoting anthocyanin release. However, excessive power caused strong physical effects that may have destabilized the anthocyanin molecular structure, thereby reducing extraction efficiency. Thus, 240 W was selected as the center level for the subsequent RSM optimization.

### 2.7. Establishment and Analysis of the Response Surface Model

#### 2.7.1. Model Establishment and Data Fitting

Based on the single-factor experimental results, ultrasonic power, ultrasonic time, and solid-to-liquid ratio were selected as the variables for subsequent response surface optimization, while temperature was held constant at its optimal level. This decision was based on the following rationale. The single-factor trials had already identified the optimal temperature (60 °C) at which the extraction efficiency peaked; beyond this point, the response either plateaued or declined. Therefore, we fixed the temperature at this optimum for all RSM runs, allowing us to concentrate on other critical factors that were less thoroughly characterized and more likely to exhibit synergistic interactions. Including temperature would have increased the number of experimental runs and model complexity without offering additional practical benefit, since its main effect and optimal level had already been established. The experimental response values are shown in Table 3. A multiple regression equation was obtained by fitting the relationship between ultrasonic power (A), ultrasonic time (B), solid-to-liquid ratio (C), and the anthocyanin content of BP as follows:

Y = 3.60 + 0.094A + 0.02B + 0.07C + 0.16AB + 0.04AC + 0.09BC − 0.11A^2^ − 0.16B^2^ − 0.31C^2^.

#### 2.7.2. Response Surface and Interaction Analysis

**Figure 9:**
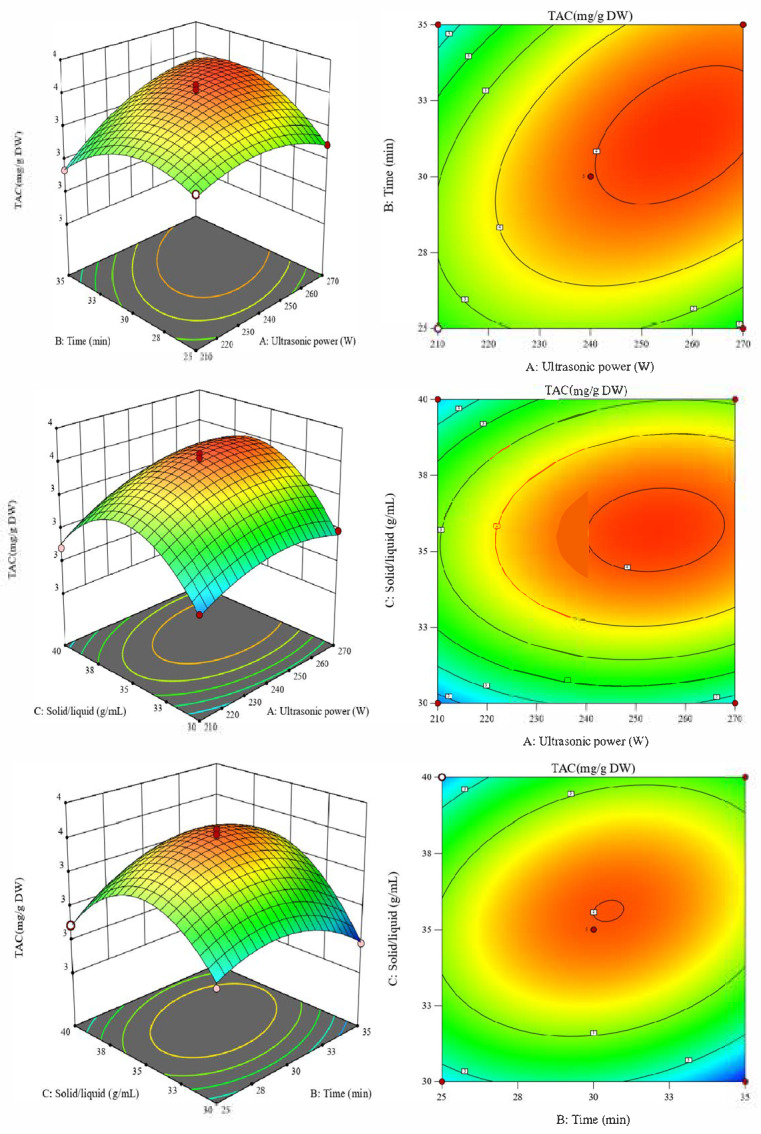
Pairwise interaction effects of factors on the TAC of BP.

The results of the analysis of variance (ANOVA) for the response surface experimental factors are shown in Table 4. The model was highly significant (*p* < 0.0001), while the lack-of-fit term was not significant (*p* > 0.05), indicating a good fit and significant main effects. According to the F-values, the order of the factors affecting the TAC of BP was A > C > B. The R^2^ and adjusted R^2^ values were 0.9860 and 0.9680, respectively, indicating that the model fits well and accurately reflects the relationship between TAC and the three factors (A, B, and C). The prediction of TAC was highly reliable with a small margin of error. Residual analysis was performed to evaluate the model adequacy. As shown in Figure S2, the residuals were randomly distributed around the zero line with no obvious trends, further confirming the reliability of the model. The three-dimensional response surfaces for the interactions among A, B, and C were steep, suggesting certain interactions between the factors, as shown in Figure 9.

### 2.8. Verification of the Optimal Extraction Process

Based on the regression model, the optimal extraction conditions were determined as follows: ultrasonic power of 260.35 W, ultrasonic time of 31.86 min, and solid-to-liquid ratio of 1:36.07 g/mL, with a predicted TAC of 3.639 mg/g. Considering the practical operability of the experimental equipment, the conditions were slightly adjusted to an ultrasonic power of 270 W, ultrasonic time of 30 min, and solid-to-liquid ratio of 1:35 g/mL, which served as the final optimized conditions. Under these adjusted conditions, the measured TAC was 3.5168 mg/g, close to the predicted value (relative error of 3.4%), which verified the reliability of the optimization. Moreover, this value was significantly higher than those obtained with 70% ethanol (3.1962 mg/g) and pure water (1.9137 mg/g), further demonstrating the superiority of the DES-UAE method.

### 2.9. Comparison of Different Extraction Methods

**Figure 10:**
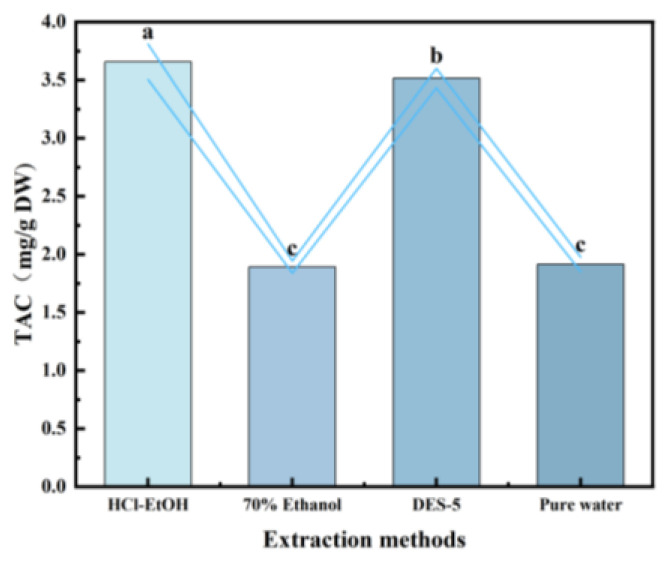
Effect of different extraction methods on TAC of BP. Different letters indicate significant differences among groups at *p* < 0.05.

As shown in Figure 10, under the same conditions, the TAC in BP extracted with HCl-EtOH was the highest (3.657 mg/g), followed by DES-5 extraction (3.5168 mg/g), while extraction with 70% ethanol and pure water gave the lowest values. The higher efficiency of HCl-EtOH may be attributed to its ability to effectively disrupt cell wall structure and stabilize anthocyanin solubilization. The high viscosity of DES-5 may affect the mass transfer rate during extraction, and its interaction with anthocyanins differs from that of conventional organic solvents. The 70% ethanol solution can effectively solubilize anthocyanins due to its low polarity and penetration capability, but its ability to damage cell walls is weaker because of the absence of a strong acid. Pure water, with its high polarity, significantly reduces extraction efficiency and also limits its ability to inhibit anthocyanin degradation in the aqueous phase. It is worth noting that although HCl-EtOH exhibited a slightly higher TAC, this solvent system is primarily designed as a standard detection method for anthocyanin quantification rather than as a viable option for industrial-scale applications. Its strong acidity (pH < 1) is not only corrosive to equipment but may also cause severe hydrolysis or structural degradation of other bioactive components in the pomace, such as pectin, dietary fiber, and residual proteins, thereby limiting the high-value utilization of the extracted residue. In contrast, despite a marginally lower TAC, DES-5 achieved comparable extraction efficiency to HCl-EtOH (approximately 96%), while its mild acidity, low toxicity, and biodegradability make it a more sustainable solvent with comparable extraction performance [33,34].

### 2.10. SEM Analysis

**Figure 11:**
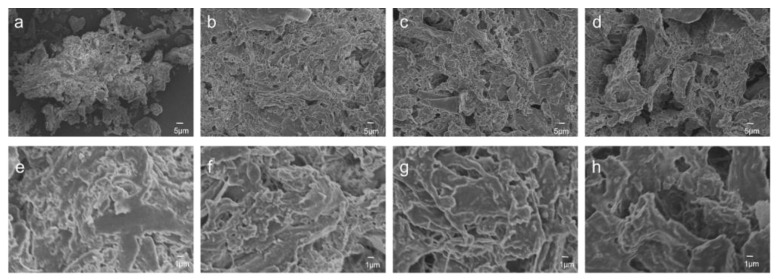
Scanning electron microscopy analysis of BP before and after DES extraction of anthocyanins. (**a**,**e**) SEM images of raw BP at different magnifications. (**b**,**f**) SEM images of BP after extraction with pure water. (**c**,**g**) SEM images of BP after extraction with 70% ethanol. (**d**,**h**) SEM images of BP after extraction with DES-5.

Since the HCl-EtOH system was only used as a reference method for the quantitative determination of anthocyanins and could not be applied to industrial food extraction and resource recycling of pomace due to its strong corrosivity, only three extraction solvents with practical green application potential, namely pure water, 70% ethanol and the optimal DES-5, were selected for SEM characterization to explore the effect of different solvents on the microstructure of pomace. SEM images of the original BP and the residues after extraction were captured at magnifications of 10,000× (scale bar: 1 μm) and 2000× (scale bar: 5 μm) (Figure 11). As shown in Figure 11a, the original BP exhibited a tightly arranged, continuous, and relatively flat surface, with no significant damage to the overall integrity. The BP extracted with pure water (Figure 11b) retained a structure most similar to that of the original sample, remaining largely intact with only slight swelling due to water absorption and a few small cracks. The BP extracted with 70% ethanol (Figure 11c) retained its overall outline but appeared significantly wrinkled and partially collapsed, with loosely connected cells and visible gaps or shrinkage-induced wrinkles. In contrast, the BP extracted with DES-5 (Figure 11d) showed the most pronounced structural changes, with the surface appearing collapsed, extensively wrinkled, and perforated, accompanied by uneven erosion. This observation indicates that DES-5 treatment substantially increased the specific surface area and porosity of the BP. Such structural enhancement facilitates the penetration of the extraction solvent into the inner cellular matrix, thereby promoting the dissolution and release of anthocyanins from the solid matrix.

### 2.11. Determination of Antioxidant Activity

**Figure 12:**
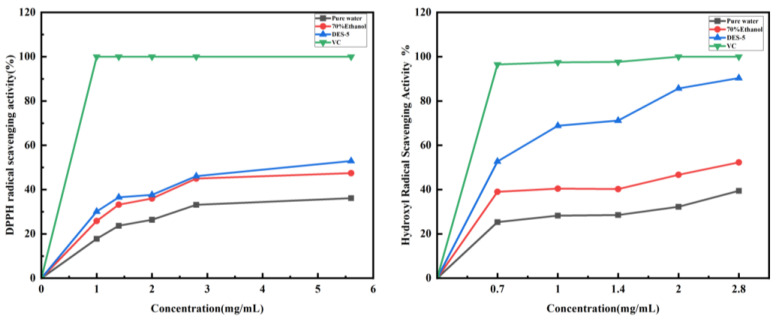
DPPH and hydroxyl radical scavenging rates of BP extracts obtained with pure water, 70% ethanol, and DES-5.

The antioxidant activities of BP extracts obtained with pure water, 70% ethanol, and DES-5 were evaluated using DPPH radical scavenging and hydroxyl radical scavenging assays, with Trolox as the positive control. As shown in Figure 12, the DPPH and hydroxyl radical scavenging rates followed a clear trend: the DES-5 extract exhibited the highest activity, followed by the 70% ethanol extract, while the pure water extract showed the lowest activity. The antioxidant activity of pure DES-5 (without BP extraction) was also measured as a blank control. The pure DES-5 exhibited negligible radical-scavenging activity, confirming that the observed antioxidant capacity of the DES-5 extract is primarily attributed to the extracted compounds from BP rather than the solvent itself.

For the FRAP assay, the values of the three extracts were significantly different from each other as shown in Table 5. Consistent with the radical scavenging assays, the DES-5 extract showed the highest FRAP value, followed by 70% ethanol, and pure water with the lowest. It should be noted that although anthocyanins are widely regarded as important contributors to antioxidant activity, their content was not significantly correlated with the antioxidant capacity observed in this study. It may be attributable to the presence of multiple other phenolic compounds in the BP extract, which also exhibit radical-scavenging properties and collectively contribute to the overall antioxidant effect. Accordingly, the measured antioxidant performance should not be exclusively ascribed to anthocyanins.

## 3. Materials and Methods

### 3.1. Raw Materials and Chemicals

Choline chloride (≥98%), betaine (≥98%), DL-malic acid (≥98%), tartaric acid (≥98%), citric acid (≥98%), 1,4-butanediol (≥98%), acetic acid (≥98%), malonamic acid (≥98%), sodium chloride (≥98%), ethanol (≥98%), TPTZ (≥98%), salicylic acid (≥98%), iron(II) sulfate heptahydrate (FeSO_4_·7H_2_O, ≥98%), and iron(III) chloride hexahydrate (FeCl_3_·6H_2_O, ≥98%) were obtained from Shanghai Bio-Engineering Co. (Shanghai, China). A DPPH assay kit was purchased from Solarbio (Beijing, China). All other reagents used were of analytical grade. An ultrasonic cleaner (Kunshan Ultrasonic Instruments Co., Ltd., Kunshan, China), a microplate reader (Detie Instruments Co., Ltd., Nanjing, China), and a high-speed centrifuge (Sigma Laborzentrifugen GmbH, Osterode am Harz, Germany) were purchased from the respective manufacturers. The BP used in this study was produced in 2025 by Tonghua Heyuan Blueberry Base (Tonghua, China), and was subsequently frozen, milled, and stored at −18 °C.

### 3.2. Preparation of DES Solution

The DES solution was prepared following the method described by D. R. et al. [35]. As shown in Table 2, choline chloride/betaine was mixed with different hydrogen bond donors in proportion using a magnetic stirring hotplate at 80 °C and 600 rpm for 30–45 min. The mixture was then cooled to room temperature (approximately 30 °C) and inspected for crystals. The absence of crystals indicated successful DES preparation. The DES solution was subsequently mixed with deionized water at a fixed liquid-to-solid ratio to obtain a 30% DES solution. This helps to reduce the viscosity of the DES solution, thereby facilitating mass transfer and improving extraction efficiency.

### 3.3. DESs Solvent Screening

BP powder (0.40 g) was accurately weighed. The ratio of BP powder to DESs was fixed at 1:25 (g/mL). Extraction was performed using ultrasonication at 40 °C and 300 W for 30 min. The extraction efficiency of different DESs (with a water content of 30%) was compared. The DES with the highest extraction efficiency was selected as the optimal solvent for subsequent anthocyanin extraction.

### 3.4. Characterization of DESs

The infrared spectrometer (NicoltlS10, Thermo Fisher, Waltham, MA, USA) was used to measure the generation of DESs and their corresponding hydrogen bond between HBA and HBDs. The DESs and liquid HBDs were applied onto KBr films, while the solid HBDs and HBA were ground together with KBr to form powder films. Absorption spectra were collected in the range of 400–4000 cm^−1^. The pH values of the seven DES systems (30% water, *v*/*v*) were measured using a pH meter at room temperature. All measurements were performed in triplicate.

### 3.5. Molecular Simulation Calculations

#### 3.5.1. COSMO-RS Calculations

The COSMO-RS model was used to predict the thermodynamic properties and perform σ-profile analysis. Quantum chemical calculations were performed using Gaussian 16(Revision C.01), and solubility predictions were conducted with COSMOtherm 2021 using the BP_TZVPD_FINE_18 parameterization at 25 °C. For the COSMO-RS calculations, cyanidin-3-O-glucoside (C3G) was selected as the representative anthocyanin. This choice was based on two considerations: (i) C3G is one of the most abundant anthocyanins in blueberries [36], and (ii) it is frequently used as a reference compound in anthocyanin extraction studies [37]. It should be noted that BP contains a complex mixture of anthocyanins, including derivatives of delphinidin, petunidin, peonidin, and malvidin, and that the use of a single representative compound may not fully capture the behavior of all anthocyanin species present in BP. Nevertheless, C3G provides a reasonable thermodynamic basis for solvent screening, and the trends observed are expected to be qualitatively applicable to other structurally similar anthocyanin derivatives. First, the molecular structures of C3G and the DES components (HBD and HBA) were optimized at the B3LYP/6-31G(d) level with D3 dispersion correction, followed by frequency analysis to confirm the absence of imaginary frequencies. DES clusters were constructed according to the experimental molar ratios by combining the optimized HBD and HBA monomers. Initial complex conformations were generated through conformational searching using OpenBabel 3.1.1 to screen low-energy hydrogen-bonding matching conformations, which were then re-optimized at the B3LYP/6-31G(d) + D3 level with frequency analysis to obtain stable DES clusters. For solute-DES complexes, the solute molecule was placed at potential interaction sites around the DES cluster, and the optimal orientation was selected via conformational searching, followed by optimization and frequency analysis. Subsequently, COSMO files for all optimized structures were generated at the B3LYP/def2-TZVP level using Gaussian 16 and used for COSMOtherm predictions with the experimental DES molar ratios as input to evaluate the dissolution capacity of different DESs for the solute. Based on the σ-profile, the hydrogen bond acceptor (HBA) region was defined as σ < −0.0082 e/Å^2^, the hydrogen bond donor (HBD) region as σ > +0.0082 e/Å^2^, and a hydrogen bond matching scoring system was established.

#### 3.5.2. IGMH Analysis

A cluster model of the complex formed between C3G and the DES was constructed and geometrically optimized at the DFT-D3/def2-TZVP level. The IGMH (Independent Gradient Model based on Hirshfeld partition) method was used to analyze weak interactions, and isosurface maps were generated using Multiwfn Multiwfn 3.8 software. Green isosurfaces represent attractive interactions (mainly hydrogen bonds), while blue isosurfaces represent repulsive interactions (steric hindrance).

#### 3.5.3. ESP Electrostatic Potential and Population Analysis

At the same theoretical level, the molecular surface electrostatic potentials (ESP) of C3G and the DES components were calculated to identify the most negative (nucleophilic) and most positive (electrophilic) regions. Hirshfeld population analysis was performed to obtain partial atomic charges of key interacting atoms and to evaluate hydrogen bond strength.

### 3.6. Optimization of DES

The best DES configuration was selected with a water content of 30% and a molar ratio ranging from 1:1 to 1:4. Subsequently, 0.40 g of BP powder was weighed and mixed with the selected DES at a ratio of 25 g/mL. Ultrasonic extraction was performed at 40 °C, 40 kHz, and 300 W for 30 min. The resulting extract was transferred to a centrifuge tube and centrifuged at 8000 rpm for 10 min.

The optimal DES configuration was selected with water content ranging from 20% to 60%. BP powder (0.40 g) was weighed and mixed with the DES at a ratio of 1:25 (g/mL). Ultrasonic extraction was performed at 40 °C, 40 kHz, and 300 W for 30 min. The resulting extract was transferred to a centrifuge tube and centrifuged at 8000 rpm for 10 min.

### 3.7. Determination of Total Anthocyanin Content (TAC)

According to the method described by Aramwit et al. [38].100 μL of blueberry anthocyanin extract was diluted 10-fold with distilled water. Then, two 40 μL aliquots of the diluted extract were taken, and each was further diluted 5-fold with buffer solutions at different pH values. After thorough mixing, the tubes were incubated at room temperature in the dark for 30 min. The absorbance was measured at 520 nm and 700 nm, and the TAC (Y) was calculated using the pH differential method as follows:

A = (A520 nm − A700 nm) pH1.0 − (A520 nm − A700 nm) pH4.5(1)

Y = (A × MW × DF × V)/(ε × L × m)(2)

where, V is the total volume of the blueberry extract (mL); DF is the total dilution factor; MW is the molecular weight of cyanidin-3-glucoside (449.2 g/mol); ε is the molar extinction coefficient of cyanidin-3-O-glucoside (26,900 L/(mol·cm)); L is the path length (1 cm); and m is the initial mass of BP powder (g).

### 3.8. Single-Factor Designs

To ensure the feasibility of the experiment, only the key factors affecting the extraction efficiency of the target compounds were considered. A single-factor design was adopted to evaluate the effects of four independent variables on anthocyanin content: solid-to-liquid ratio (1:5, 1:15, 1:25, 1:35, and 1:45 g/mL), ultrasonic time (20, 25, 30, 35, and 40 min), ultrasonic temperature (30, 40, 50, 60, and 70 °C), and ultrasonic power (180, 210, 240, 270, and 300 W). The water content of DES-5 was maintained at 40%, and 0.40 g of BP powder was used per extraction. In each run, one variable was adjusted while the other three remained unchanged. Each experimental condition was carried out in triplicate.

### 3.9. Optimization of the Procedure by RSM

Based on the findings from the single-factor experiments, the Box-Behnken design of response surface methodology (RSM) was applied to determine the key factors—ultrasonic power, ultrasonic time, and solid-to-liquid ratio—along with their coded levels (−1, 0, +1), as presented in Table 6.

### 3.10. Comparison of Different Anthocyanin Extraction Methods

Four different solvent systems were used for anthocyanin extraction: hydrochloric acid-ethanol (HCl-EtOH) [39], 70% ethanol [40], pure water, and DES-5. The HCl-EtOH solution was prepared by diluting 8 mL of concentrated hydrochloric acid (37%, *w*/*w*) with anhydrous ethanol to a final volume of 1000 mL (approximately 0.1 mol/L HCl in ethanol). After extraction and centrifugation, the anthocyanin content of the supernatant was measured.

### 3.11. Scanning Electron Microscope (SEM)

The BP morphology was analyzed using a field emission scanning electron microscope (Thermo Fisher Scientific Verios, G4 UC, Waltham, MA, USA) to evaluate the impact of different solvents on sample structural characteristics. Approximately 10 mg of each sample was freeze-dried and affixed to an SEM sample holder. To improve conductivity, a thin platinum coating was applied to the sample surfaces. The prepared samples were then placed in the SEM chamber and scanned using an electron beam to capture topographical details. Imaging was conducted at an accelerating voltage of 5 kV and magnifications of 10,000× and 2000×.

### 3.12. Antioxidant Activity Assays

For the DPPH radical scavenging assay, a commercial kit (Solarbio, BC475, Beijing, China) was used following the manufacturer’s instructions. The sample solution was diluted to different concentrations. In a 96-well plate, 10 μL of each diluted sample was mixed with 190 μL of DPPH working solution, and the mixture was incubated in the dark at room temperature for 30 min. The absorbance was measured at 515 nm (A_1_). For the control, 10 μL of the sample was mixed with 190 μL of absolute ethanol to obtain A_2_. A blank (190 μL DPPH working solution + 10 μL extraction buffer) was also prepared. The DPPH radical scavenging rate was calculated as:

D% = [A_0_ − (A_1_ − A_2_)]/A_0_ × 100% (3)

where A_0_ is the absorbance of the blank. Vitamin C was used as a positive control.

The hydroxyl radical scavenging activity was determined according to the method of Demircan et al. [41]. Briefly, 1 mL of the sample was placed in a 10 mL centrifuge tube, followed by sequential addition of 1 mL FeSO_4_ (6 mmol/L) and 1 mL H_2_O_2_ (6 mmol/L). After thorough mixing, the mixture was allowed to stand in the dark for 10 min. Then, 1 mL of salicylic acid-ethanol solution (6 mmol/L) was added, mixed, and incubated in the dark at 37 °C for 30 min. The absorbance was measured at 510 nm as A_m_. When H_2_O_2_ was replaced with distilled water, the absorbance was recorded as A_0_; when the sample was replaced with distilled water, the absorbance was recorded as A_n_. Three replicates were performed for each sample. The hydroxyl radical scavenging rate (R-OH) was calculated as:

R-OH = [1 − (A_m_ − A_0_)/A_n_] × 100%. (4)

Vitamin C was used as the positive control.

The ferric reducing antioxidant power (FRAP) assay was performed following a previous method [42]. The FRAP working solution was prepared by mixing 0.3 mol/L acetate buffer (pH 3.6), 10 mmol/L TPTZ, and 20 mmol/L FeCl_3_ in a ratio of 10:1:1 (*v*/*v*/*v*). Then, 30 μL of the sample extract and 265 μL of FRAP working solution were mixed and incubated at 37 °C for 30 min. The absorbance was measured at 593 nm. A standard curve was constructed using vitamin C (VC) as the reference, with VC concentration (mg/mL) as the abscissa and absorbance as the ordinate. The standard curve equation was y = 21.917x + 0.0059 (R^2^ = 0.9992). The FRAP value was expressed as mg VC equivalent per gram of dry sample (mg VCE/g) and calculated as follows:

FRAP = (ρ × N × V)/m(5)

where ρ is the VC equivalent concentration in the sample solution (mg/mL), N is the dilution factor, V is the total volume of the extract (mL), and m is the sample mass (g).

## 4. Conclusions

In this study, choline chloride–lactic acid (DES-5, 1:2) was identified as an effective solvent for anthocyanin extraction from BP, with the lowest ln(γ) value among the seven DESs evaluated via COSMO-RS calculations. The optimal conditions for DES-5-synergistic ultrasonic extraction (DES-5-UAE) were achieved at an ultrasonic power of 270 W, ultrasonic time of 30 min, solid-to-liquid ratio of 1:35 g/mL, and water content of 40%. UAE facilitated the release of anthocyanins by disrupting the surface structure of BP, as confirmed by SEM analysis, while COSMO-RS analysis further evidenced the enhanced solute–solvent interactions. In vitro antioxidant assays (DPPH, hydroxyl radical scavenging, and FRAP) confirmed that the DES-5-UAE extract exhibited significantly stronger antioxidant activity compared with 70% ethanol and pure water extracts.

In addition, under identical extraction conditions, HCl-EtOH yielded a slightly higher TAC than DES-5 (3.657 mg/g vs. 3.5168 mg/g), whereas DES-5, with its green attributes including low volatility, non-flammability, reduced toxicity, biocompatibility, and potential recyclability, remains one of the most promising extraction solvents. Meanwhile, it should be acknowledged that a limitation of the present study is the absence of DES solvent recovery, which is a critical factor for future industrial-scale applications. Consequently, subsequent research should focus on developing back-extraction or regeneration strategies to facilitate the practical implementation of DES-based extraction technology.

## Figures and Tables

**Table 1:** Hydrogen-bonding matching scores of the seven DESs.

DES	Composition	HB_Score	ln(γ)	Rank
DES-5	ChCl/LA	9.6	−8.197	1
DES-3	ChCl/BDO	9.2	−7.715	2
DES-4	ChCl/CA	9.1	−7.177	3
DES-1	ChCl/DL-MA	8.4	−5.388	4
DES-7	Bet/LA	7.8	−4.964	5
DES-2	ChCl/OA	6.8	−4.376	6
DES-6	Bet/PTSA·H_2_O	4.0	−4.298	7

**Table 2:** Composition and pH values of the seven DES systems.

Serial Number	Solvent Abbreviation	HBA	HBD	Molar Ratio	pH
DES-1	ChCl-MaA	Choline chloride	DL-malic acid	1:1	0.76 ± 0.01
DES-2	ChCl-OxA	Choline chloride	Anhydrous oxalic acid	1:1	0.04 ± 0.06
DES-3	ChCl-BDO	Choline chloride	1,4-Butanediol	1:3	4.24 ± 0.07
DES-4	ChCl-CitA	Choline chloride	Citric acid	1:1	0.63 ± 0.06
DES-5	ChCl-LA	Choline chloride	Lactic acid	1:2	1.22 ± 0.02
DES-6	Bet-PTSA	Betaine	p-Toluenesulfonic acid Monohydrate	1:1	0.38 ± 0.05
DES-7	Bet-LA	Betaine	Lactic acid	1:1	3.69 ± 0.07

Note: ChCl, choline chloride; Bet, betaine; PTSA·H_2_O, p-toluenesulfonic acid monohydrate. pH values were measured at room temperature with 30% water content (*v*/*v*). Data are presented as mean ± SD (*n* = 3).

**Table 3:** Box-Behnken experimental design and response values of TAC of BP.

Run	Factor			TAC (mg/g)
	A	B	C	
1	210	25	35	3.35091
2	270	25	35	3.29246
3	210	35	35	3.13661
4	270	35	35	3.54016
5	210	30	30	3.07259
6	270	30	30	3.18949
7	210	30	40	3.08373
8	270	30	40	3.37317
9	240	25	30	3.10599
10	240	35	30	2.98075
11	240	25	40	3.08929
12	240	35	40	3.33978
13	240	30	35	3.65288
14	240	30	35	3.55547
15	240	30	35	3.62366
16	240	30	35	3.55547
17	240	30	35	3.59443

**Table 4:** Analysis of variance (ANOVA) for the response surface experimental factors.

Source	Sum of Squares	Df	Mean Square	F-Value	*p*-Value
Model	0.81249	9	0.0903	54.80	<0.0001
A-Ultrasonic power	0.0706	1	0.0706	42.85	0.0003
B-Time	0.0031	1	0.0031	1.91	0.2095
C-Solid/liquid	0.0361	1	0.0361	21.90	0.0023
AB	0.0534	1	0.0534	32.40	0.0007
AC	0.0074	1	0.0074	4.52	0.0711
BC	0.0353	1	0.0353	21.43	0.0024
A^2^	0.0489	1	0.0489	29.69	0.0010
B^2^	0.1059	1	0.1059	64.27	<0.0001
C^2^	0.4017	1	0.4017	243.85	<0.0001
Residual	0.0115	7	0.0016		
Lack of Fit	0.0042	3	0.0014	0.7763	0.5652
Pure Error	0.0073	4	0.0018		
Cor Total	0.8239	16			

**Table 5:** FRAP values of BP extracts obtained with pure water, 70% ethanol, and DES-5. Different superscript letters (a, b, c) in the same column indicate significant differences (*p* < 0.05). Data are presented as mean ± SD (*n* = 3).

Extraction Solvents	FRAP
Pure water	6.5748 ± 0.2414 ^c^
70% Ethanol	23.0765 ± 0.7161 ^b^
DES-5	35.4565 ± 0.9114 ^a^

**Table 6:** Factor levels and codes for RSM design.

Factor	Levels		
	−1	0	1
Ultrasonic power/WA	210	240	270
Ultrasonic time/minB	25	30	35
Solid-to-liquid ratio/(g/mL)C	30	35	40

## Data Availability

The data that support the findings of this study are available from the corresponding author upon reasonable request.

## Supplementary Materials

The following supporting information can be downloaded at: https://www.mdpi.com/article/10.3390/molecules31132356/s1, Figure S1. FTIR spectra of the six DES systems (DES 1 to DES 4, DES 6, and DES 7) without and with 30% water addition. (FTIR spectrum of DES 5 is presented in the main text.), Figure S2. Residual analysis of the RSM model.
