# Supplementary material for: Deep Eutectic Solvent-Assisted Ultrasonic Extraction of Anthocyanins from Blueberry Pomace: Optimization, Mechanistic Insights and In Vitro Antioxidant Activity
Source: Molecules. 2026 Jul 3;31(13):2356. doi: 10.3390/molecules31132356 (PMC13362895; doi:10.3390/molecules31132356)
Supplement: Supplementary file 1 [file molecules-31-02356-s001.zip › molecules-4383973-supplementary.pdf]

Deep Eutectic Solvent-Assisted Ultrasonic Extraction of Anthocyanins from Blueberry Pomace: Optimization, Mechanistic Insights and In Vitro Antioxidant Activity

Lina Chen [1,2,†], Yue Mi [2,3,†], Xing Yang [2,3], Yunmei Ma [2,3], Chunting Zhu [2,3], Jing Xu [2,3] and Dongfang Shi [2,*]

1 College of Chemistry, Changchun Normal University, Changchun 130032, China; chenlina4321@163.com
2 Institute of Innovation Science and Technology, Changchun Normal University, Changchun 130032, China; 18097730680@163.com (Y.M.); yangyx0816@163.com (X.Y.); yunmei0205@163.com (Y.M.); zct15948840694@163.com (C.Z.); xujingl@yeah.net (J.X.)
3 College of Life Science, Changchun Normal University, Changchun 130032, China
* Correspondence: shidongfang@ccsfu.edu.cn
† These authors contributed equally to this work.

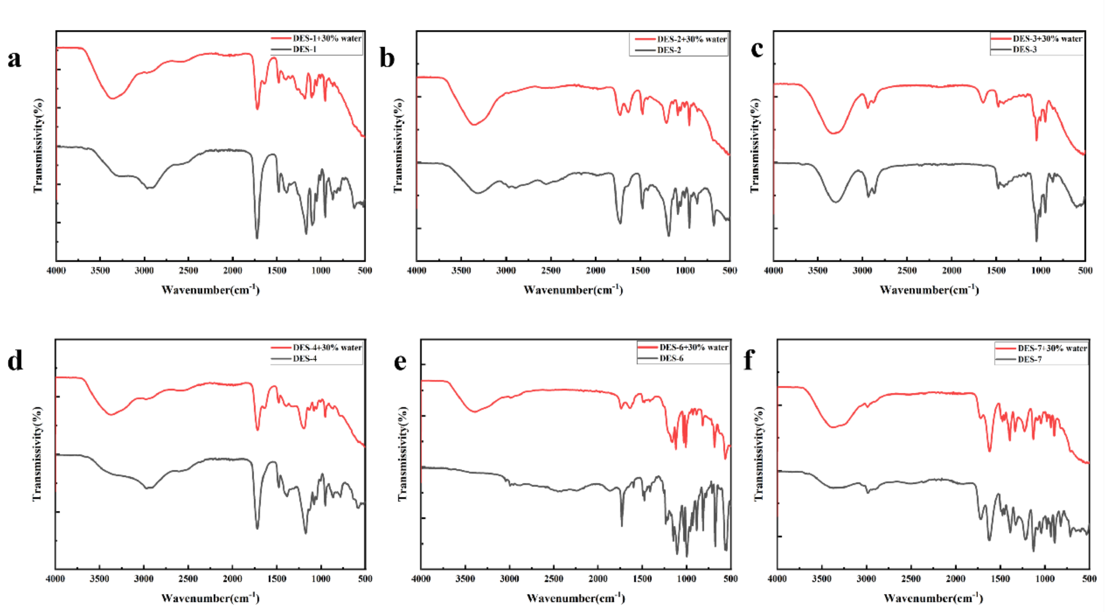

Figure S1. FTIR spectra of the six DES systems (DES-1 to DES-4, DES-6, and DES-7) without and with 30% water addition. (FTIR spectrum of DES-5 is presented in the main text.)

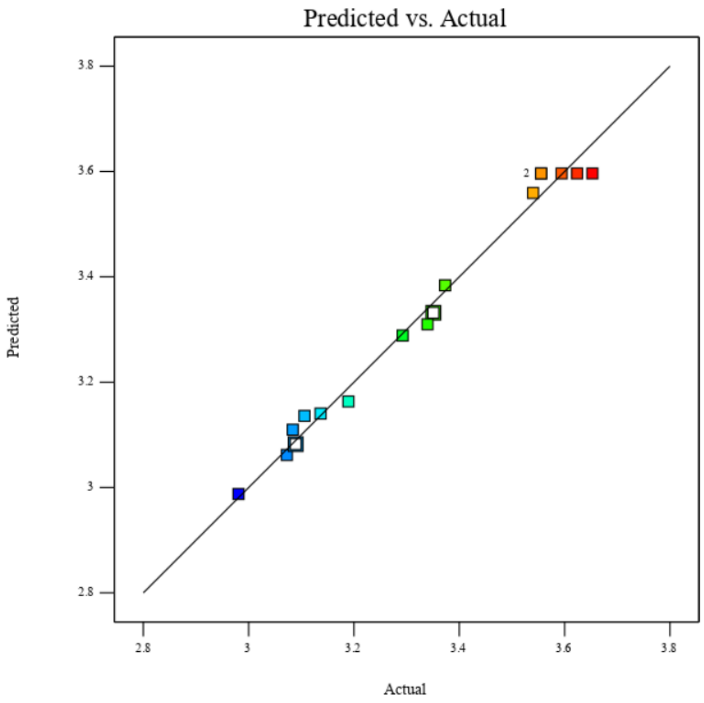

Figure S2. Residual analysis of the RSM model.
